# Association between anti-nucleophosmin and anti-cardiolipin antibodies in (NZW × BXSB)F_1 _mice and human systemic lupus erythematosus

**DOI:** 10.1186/ar1838

**Published:** 2005-10-13

**Authors:** Aurelia Lartigue, Laurent Drouot, Fabienne Jouen, Roland Charlionet, François Tron, Danièle Gilbert

**Affiliations:** 1 INSERM U519 and Institut Fédératif de Recherche Multidisciplinaire sur les Peptides, Faculté de Médecine et Pharmacie, 22 boulevard Gambetta, 76183 Rouen Cedex, France; 2 Laboratoire d'Immunopathologie Clinique et Expérimentale, CHU de Rouen, 1 rue de Germont, 76000, Rouen cedex, France

## Abstract

We showed previously that nucleophosmin (NPM), a nucleolar phosphoprotein, is recognized by sera from (NZW × BXSB)F_1 _(WB) mice, a model of systemic lupus erythematosus (SLE) and anti-phospholipid syndrome. In the present study we analysed the prevalence and kinetics of anti-NPM autoantibodies in WB mice by a solid-phase ELISA with recombinant human (rh) NPM as the antigen and showed that most male WB mouse sera had anti-NPM antibodies that were responsible for their indirect immunofluorescence staining pattern on Hep-2 cells. Anti-NPM antibodies were significantly associated with anti-cardiolipin (aCL) antibodies. This antibody profile mirrored that observed in certain human SLE sera because anti-NPM antibodies were detected in 28% of the sera from patients with SLE and were similarly associated with aCL antibodies. The demonstration that rhNPM bound to cardiolipin (CL) *in vitro *and increased the CL-binding activity of a WB-derived aCL monoclonal antibody indicates that NPM can interact with CL to form SLE-related immunogenic particles that might be responsible for the concomitant production of anti-NPM and aCL antibodies.

## Introduction

(NZW × BXSB)F_1 _(WB) mice develop an autoimmune disease whose histological and immunological manifestations resemble those of human systemic lupus erythematosus (SLE) [1]. Male WB produce anti-nuclear antibodies (ANA), including anti-deoxyribonucleic acid (DNA) autoantibodies and high levels of anti-cardiolipin (aCL) antibodies that are thought to contribute to the pathogenesis of myocardial infarction and thrombocytopenia observed in these animals [2]. aCL antibodies present in male WB mice require a plasma cofactor such as β_2_-glycoprotein I (β_2_GPI), to bind to cardiolipin (CL) and thus possess binding properties similar to those of aCL antibodies observed in the serum of patients with SLE [3,4]. Male WB mice are therefore considered an appropriate model for the secondary anti-phospholipid syndrome associated with SLE.

The precise nature of epitopes recognized by β_2_GPI-dependent aCL antibodies remains a matter of debate. Some groups consider that aCL antibodies do not recognize CL or β_2_GPI alone but bind to either a CL-β_2_GPI complex [5] or cryptic epitopes generated by their association [6]. Others think that aCL antibodies bind to β_2_GPI in the absence of CL [7]. The complexity of the interaction of anti-phospholipid antibodies with their respective antigens is further illustrated by the demonstration that β_2_GPI is not the unique cofactor involved in their binding activity. Indeed, other phospholipid-binding proteins have been described, such as prothrombin, protein C, protein S or annexin V, most of which participate in the coagulation cascade [8,9].

We previously observed that WB mouse-derived monoclonal antibodies (mAbs) selected for their capacities to react with CL in the presence of FCS also reacted with nuclear antigens, as shown by their nucleolar immunofluorescence-labelling pattern on HEp-2 cells [10]. One of these mAbs, 4B7, reacted with nucleophosmin (NPM; also known as B23), a nucleolar protein involved in the assembly and transport of ribosomes [11]. Subsequently, we showed by immunoscreening of a two-dimensional (2D) PAGE-separated HL-60 cell protein map with WB mouse sera and mass spectrometry (MS) that the males of this strain mount an ordered autoimmune B-cell response directed against various antigens, consistently including NPM [12].

These observations led us to analyse further the prevalence and kinetics of anti-NPM antibodies, their relationships with aCL antibodies in WB mouse and human SLE sera and the mechanisms that might account for the association between these two antibody populations.

## Materials and methods

### Mice and sera

Female NZW and male BXSB mice were purchased, respectively, from Bomholtgard Breeding and Research Center (Ry, Denmark) and The Jackson Laboratory (Bar Harbor, ME, USA), maintained in our animal facilities and crossbred to obtain WB offspring. Control male CD1 mice were purchased from Charles River Laboratories (Saint-Germain-sur-l'Arbresle, France). All mice were housed in the same room and fed on the same diet. Mice were bled every two months throughout their lifetime. Sera were stored at -20°C until used. Sera from (NZB × NZW)F_1 _and MRL^*lpr*/*lpr *^mice were also tested. Mouse studies were approved by the animal Ethical Committee of Normandy (ceean number 1004-27).

### Patients and sera

Serum samples were collected from 82 patients with SLE that met the criteria of the American College of Rheumatology. The serological profile of these patients was analysed for ANA by indirect immunofluorescence using HEp-2 cells, anti-DNA antibodies by the Farr assay and anti-β_2_GPI by ELISA (Varelisa β_2_-glycoprotein I IgG antibody EIA kit; Pharmacia Diagnostics, Freiburg, Germany). Serum samples were obtained from 103 healthy blood donors (agreement 31/10/2003). The study was approved by the Ethical Committee of Haute Normandie (number 99135HP).

### Indirect immunofluorescence assay

The immunofluorescence pattern of sera from WB mice and patients with SLE was determined on Hep-2 cells as described previously [10]. Inhibition experiments were performed by preincubating an anti-NPM mouse mAb (Invitrogen immunodetection, San Francisco, CA, USA), mouse and patient sera for 1 hour with 20 μg of recombinant NPM.

### Generation of mAb 4B7

Splenocytes from a four-month-old male WB mouse were fused with P3 × 63Ag8.653 myeloma cell line and the resulting hybridoma secreting mAb 4B7 was selected on the basis of the capacity of its supernatant to react with CL in ELISA. mAb 4B7 was purified as described elsewhere [10].

### Production and purification of recombinant human NPM in *Escherichia coli*

Total RNA was extracted from HL-60 cells by using TRIzol reagent (Invitrogen, Eragny, France). The NPM cDNA was obtained by transcription of oligo(dT) primer RNA with Moloney murine leukaemia virus reverse transcriptase (Invitrogen). An 879-base-pair DNA fragment was amplified from the cDNA by PCR with the primers NPM-*Nde*I (5' -GGAATTCCATATGGAAGATTCGATGGACATGGAC-3', sense) and NPM-*Bam*HI (5' -CCGGATCCTTACTTGTCATCGTCGTCCTTGTAGTCCGTACGAAGAGACTTCCTCCACTGCC-3', anti-sense) designed to create a FLAG tag followed by a stop codon and a *Bam*HI site. The purified PCR product was cloned into *Nde-Bam*HI-digested pET-15b (Novagen, Madison, WI, USA) downstream from the histidine tag. The resulting plasmid was transfected into *E. coli *BL21(DE3) pLysE (Novagen, Darmstadt, Germany). Expression was induced by incubation with isopropyl β-D-thiogalactoside (final concentration 1 mM) for 2.5 hours. *E. coli *pellets were suspended and lysed in lysis buffer (50 mM NaH_2_PO_4_, 300 mM NaCl, 10 mM imidazole, 1 mg/ml lysozyme and 1 mg/ml protease inhibitors, pH 8). The suspension was sonicated at 40% intensity for 2.5 minutes (Vibra Cell; Bioblock Scientific, Illkirch, France) and centrifuged at 7,000 *g *for 15 minutes at 4°C. The supernatant was subjected to repeated aspiration and expulsion through a fine needle to mechanically break DNA and then passed through 0.22 μm filter. Batch purification was performed with Ni^2+^-nitrilotriacetate (NTA)-Sepharose (Qiagen, Hilden, Germany). The resin was washed with buffer (50 mM NaH_2_PO_4_, 1 M NaCl, 20 mM imidazole, 20% (v/v) ethanol, pH 8) until UV absorbance at 280 nm became negative. The recombinant human nucleophosmin (rhNPM) was eluted with 50 mM NaH_2_PO_4_, 300 mM NaCl, 150 mM imidazole, pH 8. The rhNPM-enriched fractions were dialysed against water and then freeze-dried. This purification was performed for a second time with the same protocol. The protein concentration was determined with the Bradford protein assay kit (Bio-Rad Laboratories Inc., Marnes-la-Coquette, France) and the yield was about 2.5 mg per 150 ml of *E. coli *culture. Purity was controlled by SDS-PAGE analysis with a 10% polyacrylamide gel followed by western blotting with an anti-histidine-tag mAb (Sigma-Aldrich Corp., St Louis, MO, USA). rhNPM was used to detect anti-NPM antibodies, including those present in mouse sera, because they were previously shown to bind human NPM expressed by a human cell line [12] and there is 95% identity between human NPM and NO38, the murine equivalent of human NPM.

### ELISA for anti-NPM autoantibodies

High-binding plates (96-well, Microlon; Dutscher, Issy-les-Moulineaux, France) were coated with 10 μg/ml purified rhNPM in 0.05 M carbonate-bicarbonate buffer (pH 9.5) and incubated overnight at 4°C. After being washed in PBS containing 0.05% Tween, wells were blocked with PBS containing 5% (w/v) BSA for 2 hours at 23°C. Plates were washed three times with 0.05% PBS-Tween, followed by the addition to duplicate wells of mouse or human of sera, diluted 1:50 or 1:100, respectively, in diluting buffer (1% (w/v) BSA-PBS), and then incubated for 2 hours at room temperature. After being washed, biotin-conjugated goat anti-mouse IgG (Caltag Laboratories, Hamburg, Germany) diluted 1:10,000 was added, and incubated for 1 hour at room temperature. After three washes, alkaline phosphatase-conjugated streptavidin (1:10,000 dilution; Caltag Laboratories) was added and incubated for 10 minutes at room temperature. After three washes, plates were revealed with *p*-nitrophenyl phosphate (Sigma). The absorbance at 405 nm (*A*_405_) was read. Horseradish peroxidase-conjugated goat anti-human IgG (Sigma) was added and revealed with 3,3',5,5'-tetramethylbenzidine (Sigma). *A*_405 _was read. Positive mouse sera were defined as those giving an *A*_405 _reading greater than the mean value plus 2 SD of sera from 60 normal control CD1 male mice. Positive human sera were defined as those giving an *A*_405 _reading greater than the mean value plus 3 SD of sera from 103 healthy controls.

### ELISA for anti-CL antibodies

Each well of polystyrene microtitre plates (96-well; Luxlon, Nemours, France) was coated with 50 μl of bovine heart CL (10 μg/ml) in absolute alcohol. The plates were incubated overnight at 4°C to allow the ethanol to evaporate. After blocking of non-specific binding sites by incubation with 10% FCS, the plates were washed with PBS and incubated with sera diluted in FCS. The plates were washed three times followed by incubation with alkaline phosphatase-conjugated goat anti-mouse IgG (Rockland, Gilbertsville, PA, USA) or with horseradish peroxidase-conjugated goat anti-human IgG. Reactivity was determined as described above. Positive mouse sera were defined as above, whereas positive human sera were defined as those giving an *A*_405 _reading of more than 20 immunoglobulin G phospholipid international units (GPLU).

### NPM binding to CL

To determine whether NPM reacts with CL, CL-coated plates were incubated with different rhNPM concentrations for 1 hour at room temperature. After blocking of non-specific binding sites with 0.5% gelatin, plates were washed three times with PBS. Anti-histidine or anti-FLAG (Sigma-Aldrich) mAb was added and incubated for 1 hour. After three washes, alkaline phosphatase-conjugated goat anti-mouse IgG was added and revealed with *p*-nitrophenyl phosphate.

### Biacore analysis

The Biacore Biosensor system (Biacore, Uppsala, Sweden) was used to study the interaction between rhNPM and CL. Vesicles were prepared by drying 5 mg of CL under vacuum and hydrating the lipid in 1 ml of PBS as described previously [13]. The vesicles (400 μg/ml) were captured on the surface of sensor chip L1, consisting of dextran modified with lipophilic compounds. We successively injected into the system 10 μl of 20 mM CHAPS, 150 μl of vesicles and 10 μl of 10 mM NaOH to stabilize the baseline, yielding about 4,000 to 5,000 resonance units (RU) of bound antigen. Then different rhNPM concentrations (100 to 500 μg/ml) were injected and the sensor chip was regenerated with 20 mM CHAPS. Haemoglobin (Sigma) and human recombinant envoplakin produced in our laboratory were used as controls. In a second series of experiments, rhNPM was immobilized on the surface of sensor chip NTA, allowing the binding of histidine-tagged proteins. In brief, we injected 20 μl of 500 μM NiCl_2 _and then 40 μl of 200 μg/ml NPM, which yielded about 1,500 RU of antigen. The CL vesicles (400 μg/ml) were injected and the sensor chip was regenerated with 350 mM EDTA.

### Effect of rhNPM on aCL-binding activity

To examine the effect of rhNPM on aCL-binding activity, purified 4B7 mAb (0.8 mg/ml) was incubated on CL-coated plates in the presence or absence of NPM, in an ELISA as described above.

### Preparation of HL-60 cell-protein extract for 2D PAGE

Human promyelocytic leukaemia cell line (American Type Culture Collection, Manassas, VA, USA) was grown at 37°C in a humidified atmosphere (95% air, 5% CO_2_) in 50 ml of RPMI 1640 (Invitrogen, Eragny, France), supplemented with 10% FCS Sigma), antibiotics (Invitrogen) and 1 mM sodium pyruvate (Sigma). Cells were washed three times with PBS and isolated by centrifugation at 15,000 *g *for 5 minutes at room temperature. HL-60 cells (2 × 10^8^) were suspended in 10% (v/v) trichloroacetic acid, 0.12% (w/v) dithiothreitol (DTT) and stored overnight at -20°C. Cells were centrifuged at 4°C for 30 minutes at 15,000 *g*. Cell pellets were suspended again in 0.12% (w/v) DTT and kept at -20°C for 1 hour before being centrifuged. Dry pellets were suspended in lysis buffer (9 M urea, 2% (w/v) CHAPS, 1% (w/v) DTT, 2% (v/v) protease inhibitors (Sigma)) and then centrifuged at 4°C for 20 minutes at 1,500 *g*. The protein content was determined with the PlusOne 2D Quant kit (Amersham, Buckinghamshire, UK).

This lysate was subjected to 2D PAGE as previously described [12]. The immunoreactive spots were detected with human sera, diluted 1:100. After being washed, membranes were incubated with alkaline phosphatase-conjugated goat anti-human IgG (Amersham) and revealed with Nitro Blue Tetrazolium salt and 5-bromo-4-chloroindol-3-yl phosphate substrate (Roche, Meylan, France).

### Protein identification

The immunoreactive spots were excised from polyacrylamide gels with Ettan Spot Picker (Amersham) and digested by trypsin (proteomics grade; Sigma) with Ettan Digester (Amersham). Samples were analysed by matrix-assisted laser desorption/ionization-time-of-flight MS to obtain peptide mass information. Spectra obtained were compared with those registered in protein databases (SWISS-PROT and NCBInr). Data were matched against the databases with the use of the MS-Fit program (accessible through ProteinProspector).

### Statistical analysis

Absorbances of sera obtained from male and female WB mice of the same age were compared with the Mann-Whitney *U*-test. The percentages of different antibodies in the sera of patients with SLE were compared by using a χ^2 ^test. The relationship between the titres of different antibodies in mouse sera was evaluated with Spearman's correlation test.

## Results

### Male WB mouse sera frequently react with NPM

**Figure 1:**
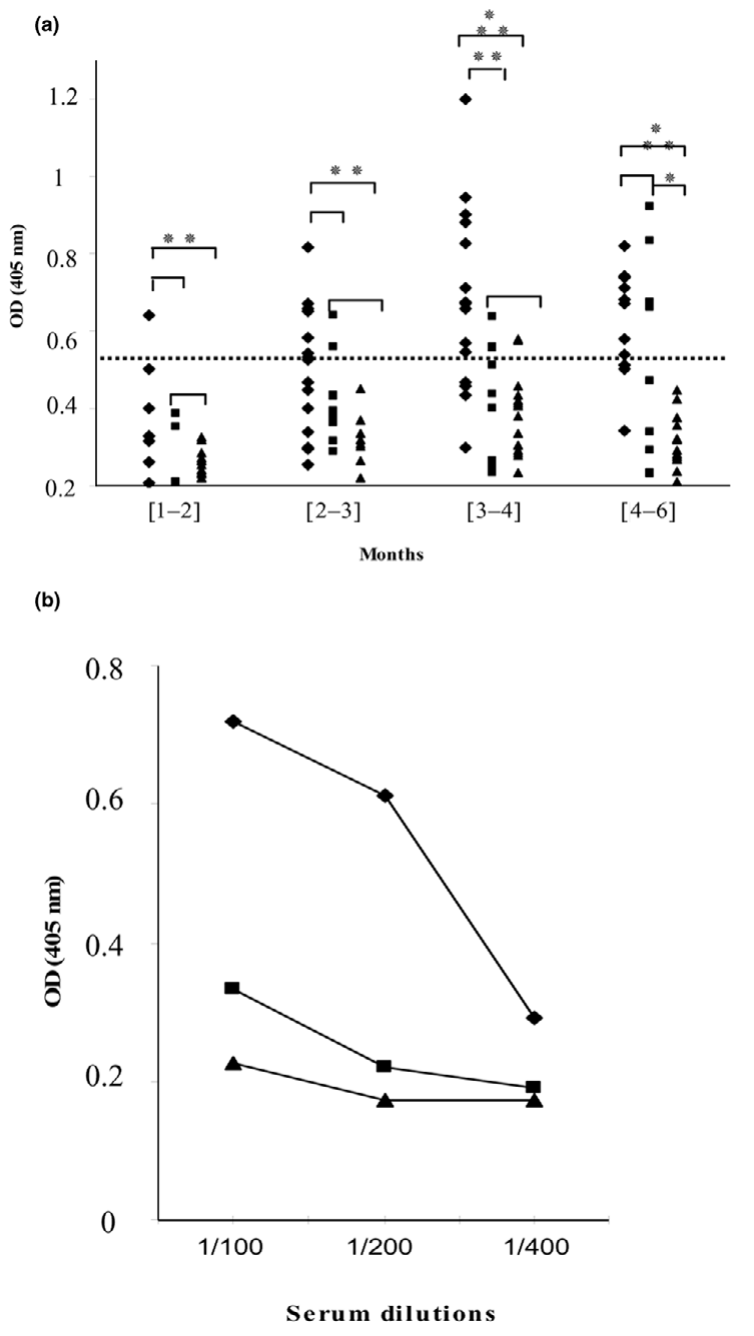
Nucleophosmin-binding activity of WB lupus-prone and control mouse sera. **(a) **Sera from male (◆) and female (■) WB mice, and from male CD1 (▴) mice, collected during months 1 to 2, 2 to 3, 3 to 4 and 4 to 6, were tested in solid-phase ELISA. The horizontal dotted line represents the cutoff value (mean *A*_405 _of controls + 2 SD). Sera with *A*_405 _< 0.522 were considered negative. (* *P *= 0.03, ** *P *= 0.01, *** *P *= 0.0001; Mann-Whitney test). **(b) **Different dilutions of male (◆) and female (■) WB and male (▴) CD1 mouse sera collected at 3.5 months were tested in ELISA.

**Figure 2:**
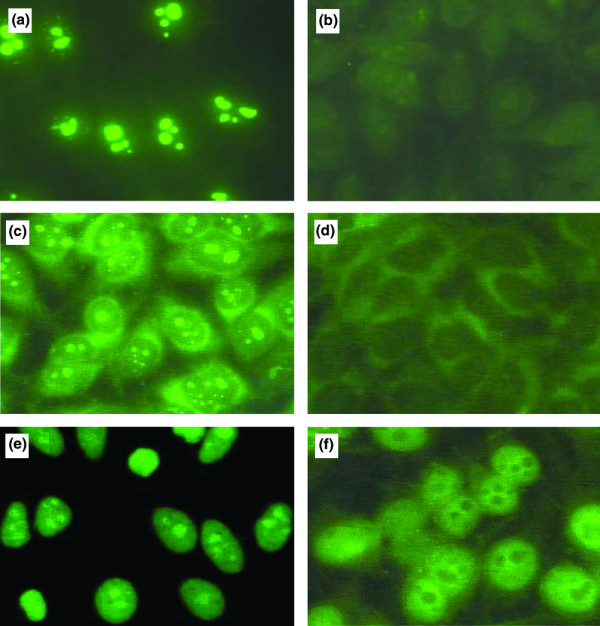
Immunofluorescence staining pattern of anti-nucleophosmin-positive WB mouse and SLE sera on HEp-2 cells. An anti-nucleophosmin (anti-NPM) mouse mAb and anti-NPM antibody-positive mouse sera gave a nucleolar pattern **(a,c)**. Anti-NPM-antibody-positive systemic lupus erythematosus (SLE) sera yielded homogeneous nuclear and nucleolar staining **(e)**. The nucleolar staining was abrogated when the mAb and sera from mouse **(b,d) **or patient **(f) **were previously incubated with 10 μg of recombinant human nucleophosmin.

To determine the prevalence of anti-NPM antibodies, sera collected from normal CD1 (*n *= 54) and WB lupus-prone mice at different times of life were analysed by solid-phase ELISA using rhNPM. As shown in Fig. 1a, anti-NPM antibodies were present early in life in male WB mice and were detected in more than 75% of sera from animals more than three months old. Anti-NPM antibodies appeared later in female WB mice (25% at 3 months), were less frequent (40% at 4 to 6 months) and gave low *A*_405 _values. The *A*_405 _of male WB sera were significantly higher than those of control mouse sera at each period (*P *= 0.01) and tested dilution (Fig. 1b), whereas female WB *A*_405 _values differed significantly from normal mouse sera only at four months (*P *= 0.03). All anti-NPM antibody-positive mouse sera, tested by immunoblotting on 2D PAGE-separated HL-60 cell-protein map, bound to the native human NPM, thereby confirming their previously reported reactivity with this nucleolar autoantigen [12]. Anti-NPM positive mouse sera gave a nucleolar staining pattern on Hep-2 cells by indirect immunofluorescence analysis similar to that observed with an anti-NPM mouse mAb (Fig. 2a,c). Preincubation of the mAb and mouse sera with recombinant NPM abrogated the nucleolar staining (Fig. 2b,d).

### NPM-binding activity in other mouse strains

We then tested WB parental strains, female NZW and male BXSB mice, but no reactivity against NPM was observed. Similarly, offspring of NZB and NZW mice also did not develop anti-NPM antibodies. In contrast, anti-NPM antibodies were present in 60% of 20 sera from MRL^*lpr*/*lpr *^mice more than three months old.

### Anti-NPM and aCL antibodies are associated in male WB mice

**Figure 3:**
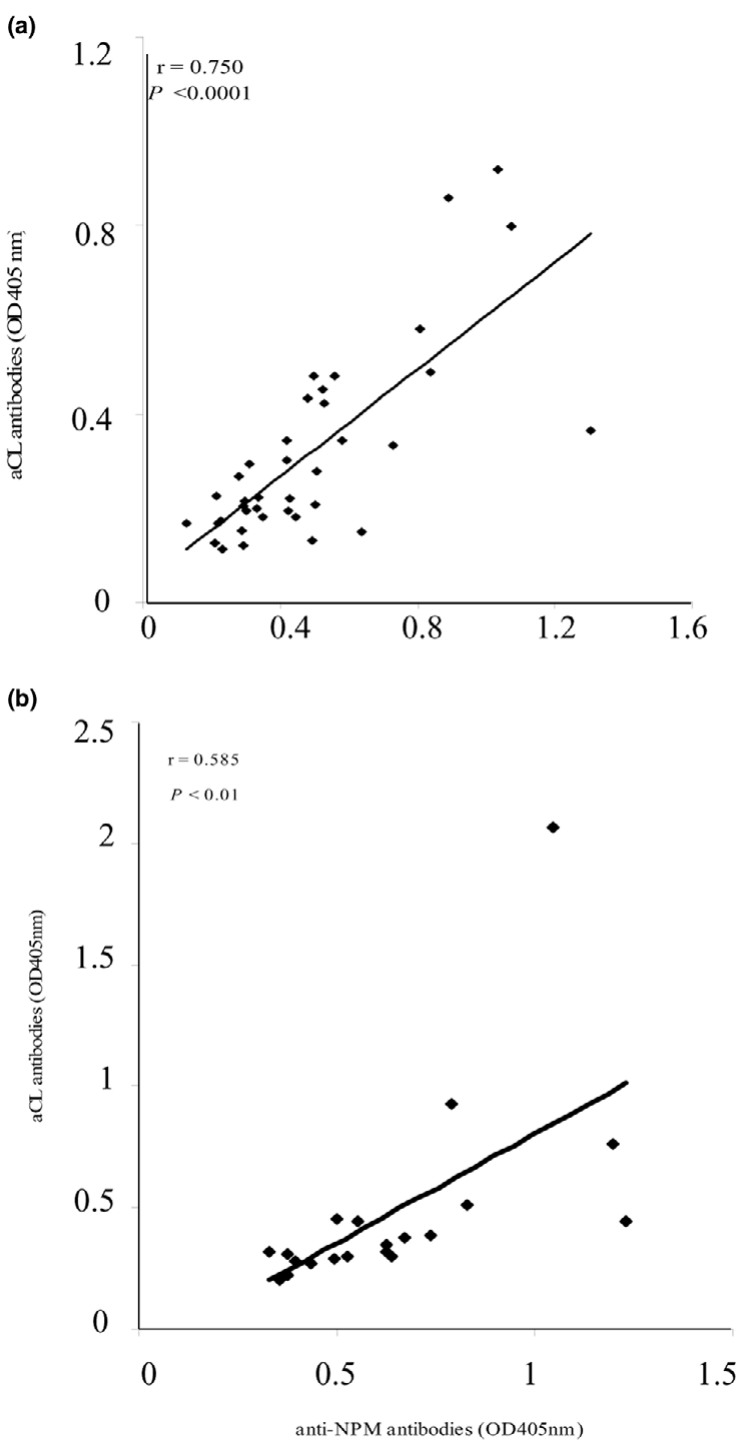
Correlation between anti-nucleophosmin and anti-cardiolipin antibodies in lupus mice. A positive correlation between anti-nucleophosmin and anti-cardiolipin antibody reactivities in 38 sera from male WB mice **(a) **and 20 sera from MRL^*lpr*/*lpr *^mice **(b) **more than 3 months old.

Because WB lupus-prone mice are also characterized by aCL antibody production, we looked for an association between anti-NPM and aCL antibodies in their sera. Indeed, anti-NPM and aCL antibodies were found to be associated in sera from three-month-old male WB mice (χ^2 ^= 18.14; *P *< 0.0001), and their *A*_405 _values were positively correlated (*r *= 0.750; *P *< 0.0001; Fig. 3a). Similarly, *A*_405 _values of anti-NPM and aCL antibodies in sera from MRL^*lpr*/*lpr *^mice more than three months old were positively correlated (*r *= 0.585; *P *< 0.01; Fig. 3b).

### Anti-NPM antibodies are present in patients with SLE and are associated with aCL antibodies

**Figure 4:**
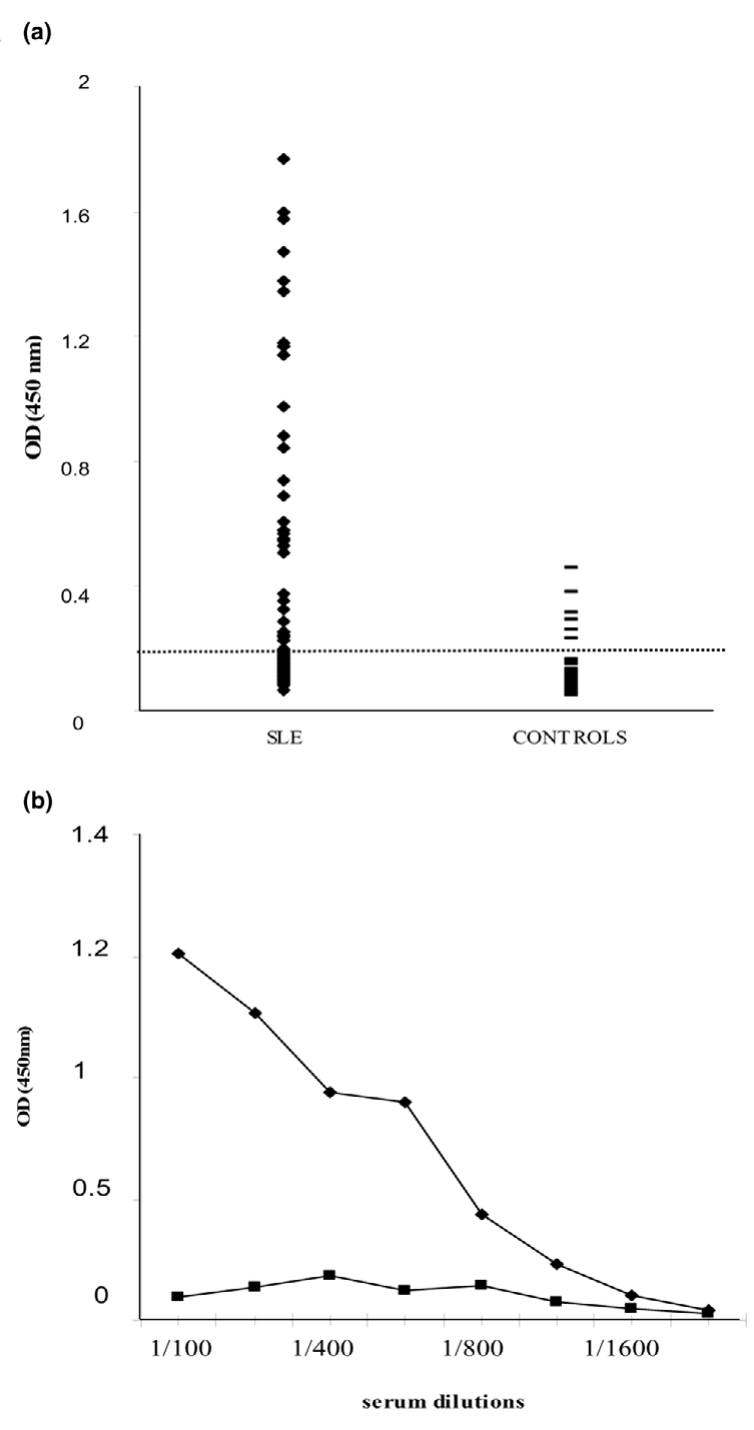
Nucleophosmin-binding activity of sera from patients with SLE. **(a) **Sera from patients with systemic lupus erythematosus (SLE) (◆) or from healthy controls (-) were analysed by ELISA using recombinant human nucleophosmin. The dotted line represents the cutoff value (mean *A*_405 _of controls + 3 SD). Sera giving an *A*_405 _< 0.286 were considered negative. **(b) **Different dilutions of serum from an SLE patient (◆) and a control (■) were tested in ELISA.

**Figure 5:**
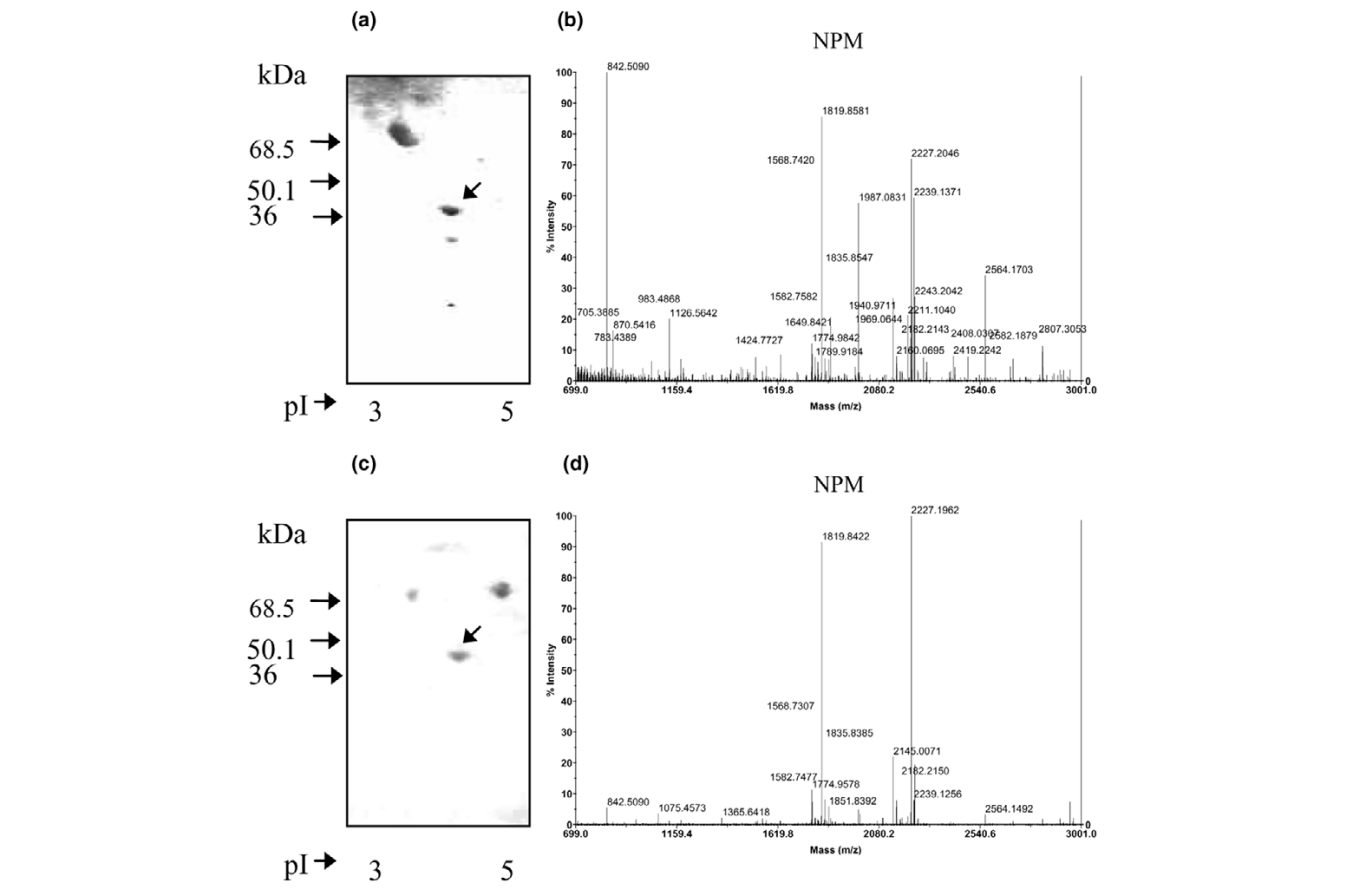
Immunoblot analyses of sera positive for anti-nucleophosmin antibody. **(a,c) **Anti-nucleophosmin antibody obtained respectively from a patient with systemic lupus erythematosus (SLE) (a) and a male WB mouse (c) using a 2D PAGE-separated HL-60 cell protein map as the substrate. **(b,d) **Mass spectra of the proteins bound by the SLE (b) and mouse (d) serum.

The demonstration that anti-NPM antibodies are frequently produced in male WB mice prompted us to search for anti-NPM antibodies in the sera of 82 patients with SLE. Indeed, 23 (28%) of these sera were positive for anti-NPM antibody (Fig. 4) and the antibodies seemed to be more frequent (although not significantly) in males than in females (5/9 versus 18/73; Table 1). These anti-NPM antibody-positive SLE sera were analysed by immunoblotting on HL-60 cell protein separated by 2D PAGE (Fig. 5a); most of them (80%) reacted consistently with a spot of molecular mass (36 kDa) and pI (4.5) that had the same coordinates as that recognized by male WB mice and was characteristic of NPM (Fig. 5c). The immunoreactive spots bound by human and mouse sera were excised and analysed by matrix-assisted laser desorption/ionization-time-of-flight MS and corresponded to NPM (Fig. 5b, d).

Anti-NPM antibody-positive SLE sera yielded homogeneous nuclear and nucleolar staining on Hep-2 cells by indirect immunofluorescence analysis (Fig. 2e). The nucleolar staining disappeared when sera were preincubated with recombinant NPM (Fig. 2f).

aCL antibodies were detected in 32 (39%) of the sera from patients with SLE. Pertinently, as in WB mice, the analysis of the distribution of anti-NPM and aCL antibodies in these SLE sera (Table 1) indicates that these autoantibodies were associated (χ^2 ^= 9.2; *P *= 0.002). In contrast, ANA, anti-DNA and anti-β_2_GPI antibodies rates did not differ significantly between anti-NPM-positive and anti-NPM-negative patients. Interestingly, of the 23 anti-NPM-positive sera, 18 (78%) did not react with β_2_GPI; conversely, of the 12 anti-β_2_GPI-aCL-positive sera, only three were positive for anti-NPM, suggesting that the presence of these two autoantibody populations is mutually exclusive.

### NPM interacts *in vitro *with CL

**Figure 6:**
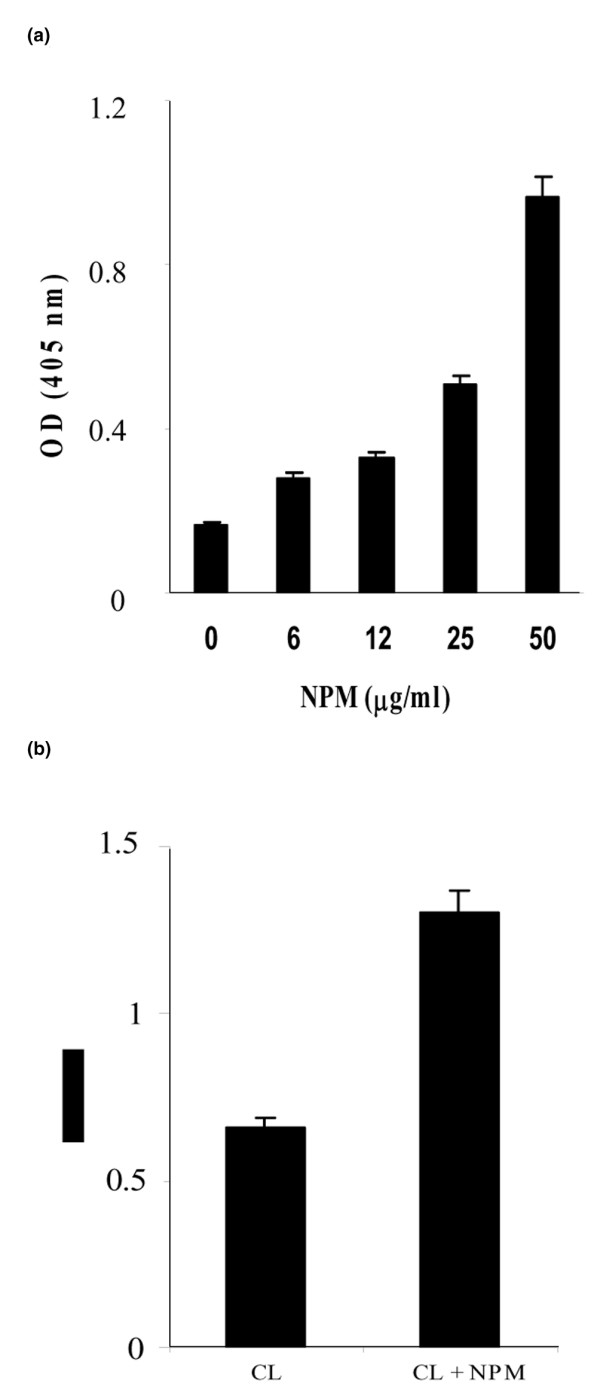
Binding of rhNPM to cardiolipin and effects of nucleophosmin on cardiolipin-binding activity of 4B7 mAb. **(a) **Cardiolipin was coated on plastic plates and incubated with various concentrations of recombinant human nucleophosmin (rhNPM). **(b) **mAb 4B7 was incubated on cardiolipin-coated plates in the absence or presence of rhNPM. Bars represent the mean *A*_405 _of duplicate experiments and error bars represent SD.

**Figure 7:**
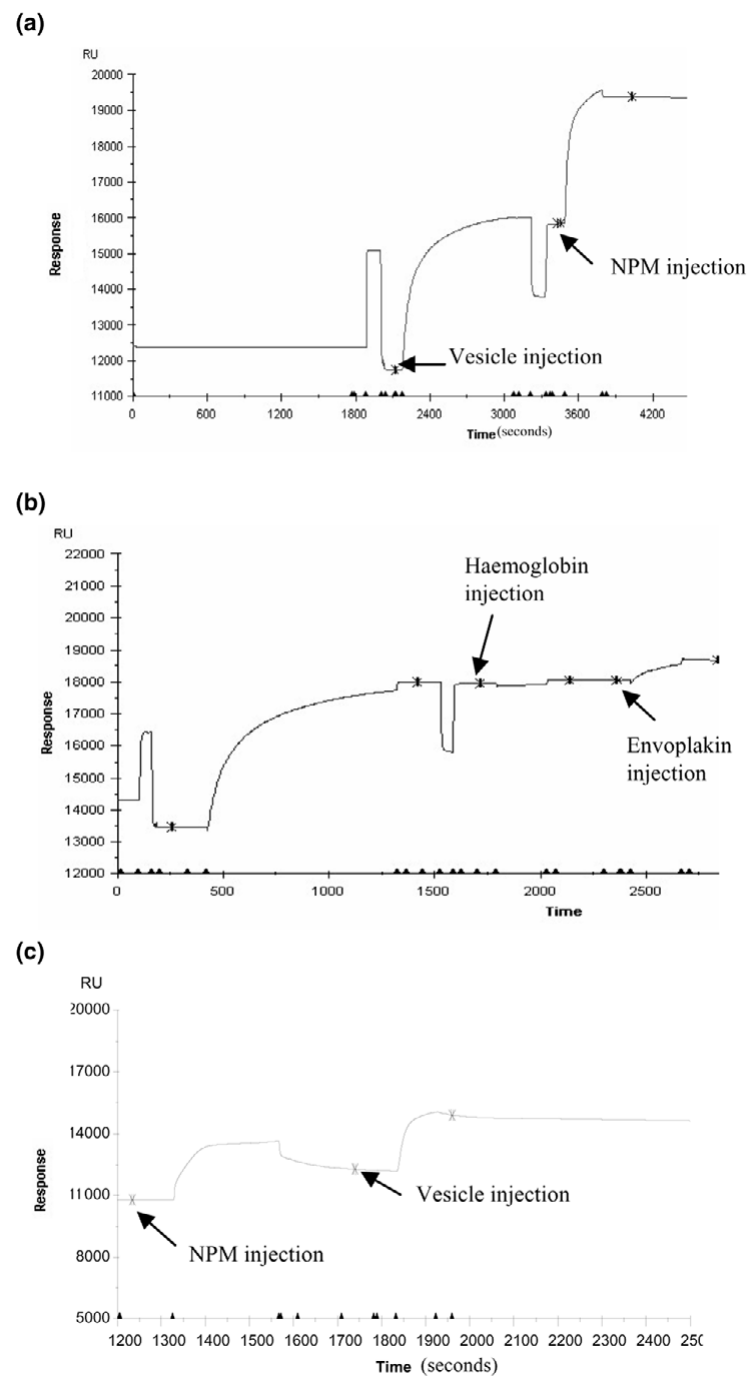
Biacore analysis of recombinant human nucleophosmin (rhNPM)-cardiolipin interaction. **(a,b) **Cardiolipin vesicles were captured on the surface of the sensor chip L1 and rhNPM (a), haemoglobin or envoplakin (b) were injected. **(c) **rhNPM was immobilized on the surface of the Ni^2+^-nitrilotriacetate sensor chip and cardiolipin vesicles were injected.

The demonstration that anti-NPM and aCL antibodies were associated in WB mice and certain patients with SLE was reminiscent of the previously described association between anti-β_2_GPI and aCL antibodies; it led us to ask whether NPM could bind to CL. CL-coated plates were incubated with increasing concentrations of rhNPM, which was revealed with an anti-histidine or anti-FLAG mAb. Figure 6a shows that NPM bound to CL-coated wells in a dose-dependent manner. This binding was confirmed by Biacore analysis. CL vesicles were captured on the surface of sensor chip L1 and NPM was injected at a concentration of 400 μg/ml. Figure 7 shows that CL vesicles bound to NPM (Fig. 7a, with a difference of 3,550 RU) but did not bind to irrelevant proteins (haemoglobin and envoplakin; Fig. 7b). Similarly, NPM captured on an NTA sensor chip bound CL vesicles, giving a response of 2,885 RU (Fig. 7c).

### NPM increases the CL binding of a murine mAb

**Figure 8:**
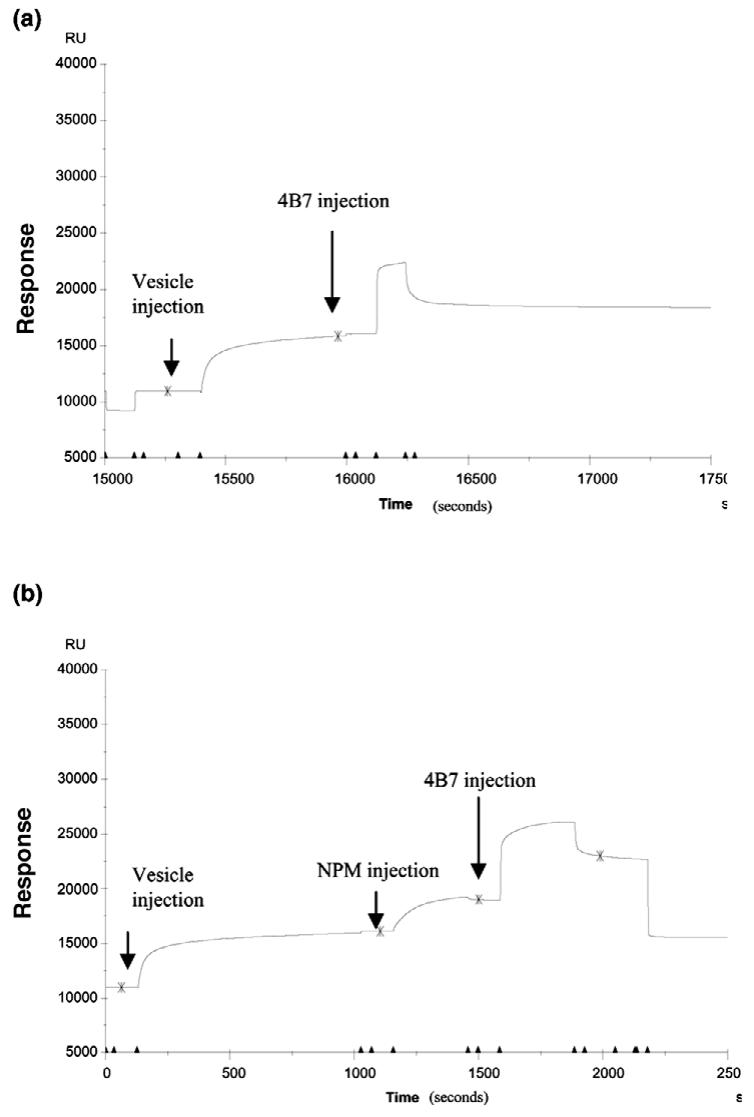
Biacore analysis of binding of 4B7 mAb to cardiolipin-nucleophosmin complexes. Cardiolipin vesicles were captured on the surface of sensor chip L1. Binding of purified 4B7 mAb to cardiolipin vesicles alone **(a) **or after the injection of recombinant human nucleophosmin **(b)**.

The demonstration that NPM could bind to CL prompted us to study the effect of rhNPM on the CL-binding activity of purified 4B7, an aCL mAb derived from a WB mouse whose serum contained both aCL and anti-NPM antibodies [10]. Indeed, in ELISA, 4B7 reactivity with CL increased markedly in the presence of NPM, which therefore acted as a cofactor (Fig. 6b). Similarly, with the use of sensor chip L1 coated with CL vesicles or CL vesicles plus NPM, the binding of 4B7 to the chip was enhanced 1.5-fold (2,578 versus 3,989 RU; Fig. 8).

## Discussion

NPM (B23) is an abundant nucleolar phosphoprotein with multiple functions: the assembly and/or transport of ribosomes [11], chaperone activities [14] and a regulatory role in cell proliferation [15,16]. NPM was previously shown to be targeted by antibodies produced in patients with either non-organ-specific autoimmune diseases [17-19] or cancer, namely hepatocellular and breast carcinoma [20,21].

By using a sensitive ELISA with rhNPM as the antigen, we showed that anti-NPM antibodies are present in most male WB lupus-prone mice and are therefore a constant feature of the antibody response in these animals: anti-NPM positivity appeared early in life, increased with age, and peaked at three to four months, before death. In contrast to our previous observations obtained by immunoblot analysis of 2D gel-separated NPM [12], this sensitive ELISA enabled us to detect these antibodies in female WB mice too, although later, less frequently and initially at lower *A*_405 _values. Pertinently, anti-NPM antibodies in male WB mice were consistently associated with aCL, and both antibody populations appeared concomitantly, as shown by sequential analyses of WB sera. Anti-NPM antibodies could also be detected in MRL^*lpr*/*lpr *^mice and again were significantly associated with aCL antibodies, which are frequently produced by this lupus mouse strain. This antibody pattern mirrored that observed in certain human SLE sera. Indeed, anti-NPM antibodies were present in 28% of our 82 patients with SLE; they were more frequent in males and were significantly associated with aCL antibodies. The association of anti-NPM and aCL antibodies was first suggested by Li and colleagues [17]. Indeed, in their analysis of 164 sera obtained from patients with various autoimmune diseases selected by the presence of anti-nucleolar antibodies, 6 had anti-NPM and aCL antibodies and SLE. However, our results, showing that anti-NPM antibodies are constantly detected in WB mice and frequently observed in patients with SLE, demonstrate that anti-NPM antibodies constitute a frequent and new marker in mouse and human lupus, establish a clear relationship between anti-NPM and aCL antibodies and finally define a subset of patients with aCL antibodies.

The association of aCL and anti-NPM antibodies in WB lupus-prone mice and patients with SLE might be explained by two different mechanisms: first, cross-reactivity due to the expression of a shared epitope by CL and NPM, or second, the ability of NPM to interact with CL to form an immunogenic complex able to induce the two antibody populations and/or a unique antibody population able to react with both NPM and CL (dual reactivity), as reported for lupus-related antigen particles [22-24]. The former hypothesis is not supported by the presence of anti-NPM-positive/aCL-negative sera and, conversely, aCL-positive/anti-NPM-negative sera and by the demonstration that anti-NPM antibodies induced by immunization of normal mice do not react with CL (data not shown); this conclusion was also reached by Li and colleagues [17], who showed that affinity-purified anti-NPM antibodies from SLE sera did not bind to CL. We therefore tested the second hypothesis, which proposed that lupus-related antigens are made of physically linked epitopes, such as DNA-histones [22] or Sm-DNA [23,24] and implies that NPM is able to bind to CL and behave as an aCL antibody cofactor. Plasmon resonance analysis of rhNPM-CL interaction clearly showed that NPM binds to CL *in vitro *to form complexes. This binding might be attributed to the functional N-terminal domain, which contains a high density of hydrophobic residues involved in chaperone activity [25]. The same technology enabled us to show that the reactivity of 4B7 mAb to NPM-CL complexes was markedly enhanced in comparison with its reactivity to either NPM or CL alone, suggesting that 4B7 is representative of mAbs exhibiting a dual specificity similar to that previously reported for certain anti-histone murine mAbs, whose binding activity is increased by DNA [22,26]. In human SLE sera, such autoantibodies probably exist but their identification remains elusive because of the polyclonal nature of sera, which may simultaneously contain monoreactive anti-NPM and anti-CL antibodies.

These results could also lead us to consider that NPM acts as an aCL cofactor, at least in this murine model of lupus. So far, several cofactors of anti-phospholipid antibodies have been described; most of them are soluble proteins involved in coagulation [27,28]. We have showed here that a nuclear autoantigen can behave as an aCL cofactor. This observation raises important questions: is NPM the unique nuclear autoantigen acting as an aCL cofactor? When and where does NPM interact with CL to form an immunogenic complex able to initiate antibody responses to both proteins? NPM is an abundant nuclear protein [29] that can translocate to the cytoplasm [30]. Recently, it was found to be localized in the cell membrane and to be a component of the fructose lysine-specific receptor expressed by monocyte-like cell lines [31]. Thus, NPM could well interact with anionic phospholipids at the cell membrane to form a typical lupus-related immunogenic complex, which could be released. Experiments are under way to determine the cellular localization of NPM in different categories of cells and during various cellular processes, such as apoptosis, which is thought to have a major role in the breakage of B cell tolerance in SLE [32]. The answer to these questions will help to explain the high frequency of anti-phospholipid antibodies in patients with SLE and to identify other nuclear autoantigens able to behave as an aCL cofactor. Another important objective is to precisely define the sensitivity and specificity of anti-NPM antibodies for lupus and to determine whether their presence is correlated with disease activity or clinical manifestations in patients with SLE. Such an analysis is under way in a large series of patients with SLE and various autoimmune diseases and will help to clarify the disease significance of this autoantibody population.

## Conclusion

In this study we show that anti-NPM antibodies constitute a frequent marker in WB and MRL^*lpr*/*lpr *^lupus-prone mice and in human SLE, establish a clear relationship between anti-NPM and aCL antibodies and define a subset of patients with aCL antibodies. The demonstration that NPM binds to CL *in vitro *and increases the CL-binding activity of a WB-derived aCL mAb indicates that NPM can interact with CL to form SLE-related immunogenic particles that might be responsible for the concomitant production of anti-NPM and aCL antibodies.

## Abbreviations

2D = two-dimensional; aCL = anti-cardiolipin; ANA = anti-nuclear antibodies; β_2_GPI = β_2_-glycoprotein I; BSA = bovine serum albumin; CL = cardiolipin; DTT = dithiothreitol; ELISA = enzyme-linked immunosorbent assay; FCS = fetal calf serum; mAb = monoclonal antibodies; MS = mass spectrometry; NPM = nucleophosmin; NTA = nitrilotriacetate; PBS = phosphate-buffered saline; PCR = polymerase chain reaction; rhNPM = recombinant human nucleophosmin; RU = resonance units; SLE = systemic lupus erythematosus; WB = (NZW × BXSB)F_1_.

## Competing interests

The authors declare that they have no competing interests.

## Authors' contributions

AL participated in the design of the study, performed the immunoanalysis and wrote the manuscript. LD participated in recombinant protein synthesis. FB provided sera from patients. RC performed mass spectrometry analysis. FT participated in the design of the study and wrote the manuscript. DG performed Biacore analysis, participated in the design of the study and wrote the manuscript. All authors read and approved the final manuscript.

## Figures and Tables

**Table 1:** Demographic and serological profiles of patients with SLE

Characteristic	SLE patients	Anti-NPM antibodies	
	(*n *= 82)	Positive (*n *= 23)	Negative (*n *= 59)
Age, years (mean ± SD)	38.3 ± 12.3	39.8 ± 14.9	37.8 ± 11.5
Females	73 (89.0)	18*	55
Males	9 (10.8)	5*	4
aCL	32 (39)	15 (65.2)**	17 (28.8)**
ANA-positive	66 (80.5)	23 (100)	43 (72.8)
Anti-DNA	46 (56)	13 (56.5)	33 (55.9)
Anti-β_2_GPI	15 (18.3)	5 (21.7)	10 (16.9)
Anti-β_2_GPI-CL+	12 (14.6)	3 (13)	9 (15.2)

aCL, anti-cardiolipin; ANA, anti-nuclear antibodies; β_2_GPI, β_2_-glycoprotein I; CL, cardiolipin; NPM, nucleophosmin; SLE, systemic lupus erythematosus. Values are *n *(%) unless indicated otherwise.

* χ^2 ^= 2.4; *P *= 0.12. ** χ^2 ^= 9.2; *P *= 0.002.
